# COVID-19 Vaccine Hesitancy among Italian University Students: A Cross-Sectional Survey during the First Months of the Vaccination Campaign

**DOI:** 10.3390/vaccines9111292

**Published:** 2021-11-07

**Authors:** Valentina Baccolini, Erika Renzi, Claudia Isonne, Giuseppe Migliara, Azzurra Massimi, Corrado De Vito, Carolina Marzuillo, Paolo Villari

**Affiliations:** Department of Public Health and Infectious Diseases, Sapienza University of Rome, 00185 Rome, Italy; erika.renzi@uniroma1.it (E.R.); claudia.isonne@uniroma1.it (C.I.); giuseppe.migliara@uniroma1.it (G.M.); azzurra.massimi@uniroma1.it (A.M.); corrado.devito@uniroma1.it (C.D.V.); carolina.marzuillo@uniroma1.it (C.M.); paolo.villari@uniroma1.it (P.V.)

**Keywords:** vaccine hesitancy, COVID-19, students

## Abstract

Achieving high levels of vaccination coverage against COVID-19 may be hindered by vaccine hesitancy. We quantified over time the prevalence of COVID-19 vaccine hesitancy among university students, investigated its determinants, and analyzed student attitudes, risk perceptions and compliance with preventive measures. The survey was administered online from 1 March to 30 June 2021. A multivariable logistic regression model was built to identify predictors of hesitancy. Overall, we collected 5369 questionnaires that were grouped into three survey periods (March, April–May, and May–June). The response rate ranged from 81.2% to 76.4%, whereas vaccine hesitancy ranged from 22% to 29%. Multivariable analysis showed that April–May participants had higher odds of hesitancy than March respondents. Other positive predictors were being male, not being a healthcare student, having a lower academic level, and not disclosing a political position. Conversely, higher levels of perceived COVID-19 severity, concern for the emergency, confidence in vaccine safety and effectiveness, and self-reported adherence to mask wearing indoors and outdoors were negatively associated with hesitancy. We found that vaccine hesitancy changed over time and in relation to several factors. Strategies aimed at increasing the students’ awareness and engagement, restoring confidence in health authorities, and limiting disinformation around the vaccines should be devised.

## 1. Introduction

The coronavirus disease 2019 (COVID-19) pandemic has ushered in a new era of immunization, reminding the world of the power of vaccines to safeguard public health [1]. Nevertheless, despite efforts to promote the vaccination campaigns, reaching a high level of vaccination coverage in individual countries, and particularly worldwide, is still far away [2]. Among the hindering factors, vaccine hesitancy may play a role [3]. It is defined as a “delay in acceptance or refusal of vaccines despite availability of vaccine services” [4], and it is a phenomenon of such concern that, already in the pre-pandemic era, it was listed by the World Health Organization (WHO) as one of the major threats to global health [5]. In recent years, the reasons for such behavior have been studied in relation to measles or influenza vaccination, both in the general population [6,7] and in specific subgroups [8,9,10], showing that they are multifaceted and culture-specific, but they can be broadly grouped into contextual, individual, group, and vaccine-specific factors [4].

While evidence on hesitancy towards SARS-CoV-2 vaccines is still evolving, the promotion of vaccination uptake faces unprecedented challenges due to the rapid development of the vaccines, the relatively new techniques used for their development, the occurrence of rare but severe adverse reactions, and the continuous changes in policy responses around the world [11]. Although there is variability in the degree of hesitancy, it is present in both low- and high-income countries and is widespread across socioeconomic, religious, and ethnic groups [12,13]. Moreover, there was a progressive decrease in vaccine acceptance between the first months of the pandemic and December 2020 [12], highlighting the importance of monitoring this phenomenon over time and analyzing its determinants in specific population subgroups so that timely and effective intervention strategies can be devised [3].

Within this context, a few researchers have investigated university students [14,15,16]. The results show that, on the one hand, students have great levels of social interaction and mobility [17] that, coupled with a disease profile that is often asymptomatic or shows few symptoms [18], may make this subgroup responsible for the spread of the virus. On the other hand, students are still in full- or part-time education and therefore may be prone to change their behavior [19], making them a good target for educational interventions. In the Italian setting, three studies have focused on young adults [20,21,22], but little is known about the evolution of student vaccination intention during the vaccine rollout. Furthermore, an analysis of their attitudes, risk perceptions, and adherence to precautionary measures is still lacking. This study aims (i) to quantify over time the prevalence of hesitancy towards COVID-19 vaccination between March and June 2021 among Italian university students and to investigate its determinants; and (ii) to analyze their attitudes, risk perceptions, and adherence to recommended precautions. This information could support policymakers in promoting COVID-19 immunization.

## 2. Materials and Methods

### 2.1. Setting and Participants

This cross-sectional study was conducted during the free SARS-CoV-2 screening campaign offered to regularly enrolled students by Sapienza University of Rome. Specifically, students who attended in-person educational activities at the main campus from 1 March to 30 June 2021 were offered to undergo an RT-PCR molecular test. This student swab service ran on Monday–Friday from 8 am to 4 pm for the first two weeks. It was then paused when the University was closed because of COVID-19 restrictions and Easter holidays, restarting again on 12 April 2021, and running Monday–Thursday with the same opening hours until the end of June. While students were waiting for their turn at the screening site, they were invited to voluntarily take part in an online survey accessible via smartphone through a QR code. Whereas it was possible to take more than one swab throughout the screening campaign, the questionnaire was available only once.

The study was performed in accordance with the World Medical Association Declaration of Helsinki. Participants were asked for their consent and were guaranteed anonymity in the information collected. The institutional ethics board of the Umberto I teaching hospital/Sapienza University of Rome approved this study (protocol 226/2021).

### 2.2. Questionnaire

The questionnaire was derived from a literature review [15,16,19,23]. It consisted of 20 questions grouped into four sections and took approximately five minutes to fill out.

The first section aimed to collect sociodemographic information: age, gender, nationality, faculty, year of study, finances (i.e., with the financial resources at your disposal, how well do you get to the end of the month?), and politics (i.e., when it comes to politics, where would you place yourself on a scale from 1 [strong left-wing] to 9 [strong right-wing]?).

The second section explored COVID-19 experience and perceptions. Specifically, one question investigated the occurrence and symptoms of a past infection, whereas the others asked students to rate from 0 (not at all) to 10 (extreme) their personal risk of COVID-19 infection (perceived susceptibility), the severity of COVID-19 disease (perceived severity), or their concern about the COVID-19 emergency.

The third section focused on COVID-19 vaccination. We asked whether they had received at least a single dose of a COVID vaccine (i.e., are you already vaccinated against COVID-19 with at least one dose?), and, since our target cohort was unvaccinated students, the questionnaire ended at this point for participants who answered yes. For students who answered no, we investigated their vaccination intention (i.e., on a scale from 0 [not at all] to 10 [definitely], how likely is that you’ll get a COVID-19 vaccine?), the main reasons for being hesitant, and the confidence in vaccine safety and effectiveness (from 0 [not at all confident] to 10 [extremely confident]). Given the scientific and political debate around COVID-19 vaccine types that arose during March 2021, beginning on 12 April 2021 (i.e., as soon as the screening campaign re-started, after the Easter holidays), two optional questions were added to the last section of this questionnaire: (i) on a scale from 0 (not at all) to 10 (definitely), how much would you like to choose which COVID-19 vaccine to take? and (ii) being able to choose, what is the main feature that you would consider in the choice of the vaccine?

The last section investigated self-reported adherence to five recommended COVID-19 precautionary measures. We asked participants to rate from 0 (never) to 10 (always) how frequently they were usually wearing (i) a community or surgical mask indoors when recommended; (ii) a FFP2 mask indoors when recommended; (iii) any face mask outdoors when recommended; we also asked how frequently they (iv) were performing hand hygiene when recommended; and (v) were respecting physical distancing during outdoor activities.

### 2.3. Statistical Analysis

Descriptive statistics were obtained using median and interquartile range, or mean and standard deviation, for continuous variables and proportions for dichotomous and categorical variables. For the purposes of this analysis, students were considered as Italian vs. non-Italian. Faculties were grouped into three categories: healthcare (e.g., medicine, nursing), science (e.g., mathematics, biology), or other (e.g., law, economics). Participants were classified into two groups according to their year of study: first- and second-year students (i.e., who started their university career during the pandemic) vs. third-year students or above. Politics was categorized into four classes: strongly left-wing (i.e., answering 1 or 2), moderate (i.e., answering from 3 to 7), strongly right-wing (i.e., answering 8 or 9), and prefer not to answer. The two questions regarding community/surgical masks and FFP2 masks were collapsed into one variable, adherence to mask wearing indoors. The highest value of self-reported adherence between the two options was considered. Questionnaires were divided into three periods according to the date of survey completion: (i) from 1 March to 12 March 2021 (i.e., before university closure); (ii) from 12 April to 9 May 2021 (i.e., during the first month of the screening campaign after university re-opening); and (iii) from 10 May to 30 June 2021 (i.e., to the end of the SARS-CoV-2 screening campaign). We chose 9 May as the second cut-off because it divided the remaining students into two groups of similar size. Vaccine hesitancy was measured as the complement to 10 of students’ intention to get vaccinated. Given the high prevalence of students that expressed no hesitancy (i.e., that answered 10/10 to the question “on a scale from 0 [not at all] to 10 [definitely], how likely is that you’ll get a COVID-19 vaccine?”), the outcome was collapsed into two levels: having no hesitancy vs. having some degree of hesitancy.

For the univariable analysis, the Kruskal–Wallis test was used to compare continuous variables across survey periods, whereas Pearson’s chi-squared test was used for dichotomous and categorical variables. A multivariable logistic regression model was built to identify predictors of vaccine hesitancy. Variables were included in the model based on expert opinion [24]. Multicollinearity was checked using as threshold a variance inflation factor of 5. The Hosmer and Lemeshow test was used to evaluate the goodness of fit of the model. As a result, the final model consisted of the following variables: survey period (categorical), age (continuous), gender (dichotomous), nationality (dichotomous), area of study (categorical), year of study (dichotomous), finances (dichotomous, i.e., having some or many financial difficulties vs. getting to the end of the month well enough or very well), politics (categorical), perceived susceptibility to COVID-19 (continuous), perceived COVID-19 severity (continuous), concern about the COVID-19 emergency (continuous), COVID-19 past infection (dichotomous, i.e., yes vs. no), confidence in vaccine safety (continuous) and effectiveness (continuous), adherence to mask wearing indoors (continuous), adherence to mask wearing outdoors (continuous), performing hand hygiene (continuous), and maintaining physical distancing (continuous). Adjusted odds ratios (ORs) and 95% confidence intervals (CIs) were calculated. A subgroup analysis was performed on students belonging to the healthcare area. The same methods and variable selection process of the main analysis were used.

All analyses were performed using Stata (StataCorp LLC, 4905 Lakeway Drive, College Station, TX 322, USA), version 17.0. A two-sided *p*-value < 0.05 was considered statistically significant.

## 3. Results

During the screening campaign, 7339 students were tested at least once for SARS-CoV-2; of these, 5784 answered the questionnaire (overall response rate: 78.8%, ranging from 81.2% in March and 76.4% in May–June). However, 415 participants had already been vaccinated with at least one dose (one student in March, 19 students in April–May, and 395 students in May–June) and therefore were excluded, giving a total of 5369 questionnaires analyzed: 1910 students in the first survey period (daily mean: 191, range 150–231), 1720 students in the second survey period (daily mean: 107, range 60–159), and 1739 students in the third survey period (daily mean: 62, range 12–128).

Respondents were aged 23.5 ± 4.5 on average (Table 1). Most participants were females (around 60%), and this proportion increased over time to almost two-thirds between May and June 2021. Only a minority of students were non-Italian, even though their prevalence became higher over time. The largest category was students enrolled in faculties not related to healthcare or science. More than half of the students had a high academic level (third year or above). Almost 50% of the participants reported that they got to the end of the month (financially) well enough, while 20% did very well, and the remaining one-third had some or more difficulties. As for politics, approximately half of the students adopted a moderate position, around 12% stated they were strongly left-wing, while only a limited number of students identified as strongly right-wing (~1%). An increasing proportion of respondents over the study period preferred not to indicate their political position.

Perceived COVID-19 severity and concern about the emergency seemed to decrease over time, likewise perceived susceptibility, which also registered lower absolute values throughout the campaign (Table 2). The prevalence of students with a past COVID-19 infection slightly increased, from almost 4% in March to 7% between May and June. During the first survey period, 78% of students showed no hesitancy in their intention to get vaccinated, but this proportion decreased throughout the campaign. The main reasons for hesitancy were (i) not considering themselves at risk of infection (around 20%); (ii) low confidence in vaccine safety or effectiveness, especially during March, when together this group represented more than 37% of the cohort, but which fell to around 25% in the other survey periods; (iii) and being aware of serious adverse reactions occurring after vaccination; the prevalence of this category doubled over time (from 13% in March to 26% in May–June), becoming the most frequent reason given. A decreasing mean confidence in vaccine safety was registered, which fell by 0.50 throughout the campaign (from 8.05 in March to 7.54 in May–June). By contrast, no meaningful change was observed in confidence in vaccine effectiveness. As for self-reported adherence to precautionary measures, mask wearing indoors scored the highest compliance (around 9 out of 10), but seemed to decline over time, in line with the decreasing levels of mask wearing overall and maintenance of physical distancing. Conversely, adherence to hand hygiene guidelines peaked during the second survey period, returning to pre-peak values immediately thereafter.

**Figure 1:**
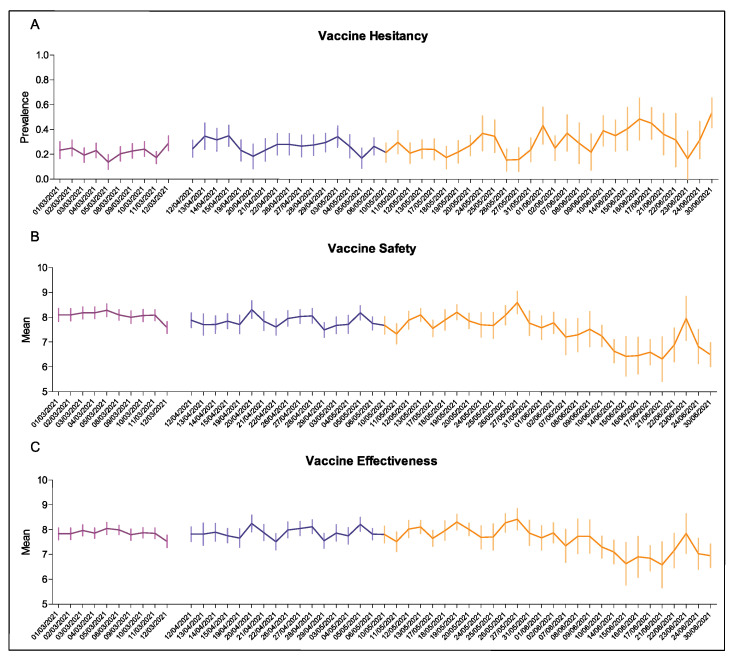
Prevalence of vaccine hesitancy (**A**), mean confidence in vaccine safety (**B**) and effectiveness (**C**) among the students surveyed from March 1st and 12 March 2021 (first period, purple line), from 12 April to 9 May 2021 (second period, blue line) and from 10 May to 30 June 2021 (third period, orange line) at Sapienza University of Rome.

Daily prevalence of vaccine hesitancy throughout the SARS-CoV-2 screening campaign is illustrated in Figure 1A, where a gradual increase is observable. Confidence in vaccine safety visibly declined over time (Figure 1B), whereas confidence in vaccine effectiveness fluctuated somewhat in the first weeks, but showed a clear drop during the last few days only (Figure 1C).

The two optional questions added to the questionnaire from 12 April are reported in Table 3. Students expressed a higher willingness to choose the vaccine in May–June compared to April–May. Main drivers of choice were personal experience with side effects (around 26%), higher efficacy (23–25%) or safety (21–22%) in trials, and the type of vaccine (20–22%). Less importance was given to the country of vaccine production (around 4%) (Table 3).

In the multivariable analysis, higher odds of vaccine hesitancy were found for students surveyed in the second period only (aOR: 1.25, 95% CI: 1.04–1.50) (Table 4). Similarly, being male, not a healthcare student, or being in a lower study year were associated with higher likelihood of hesitancy (male: aOR: 1.39, 95% CI: 1.18–1.64; science as area of study: aOR: 1.33, 95% CI: 1.08–1.64; neither healthcare or science as area of study: aOR: 1.40, 95% CI: 1.16–1.70, and first- or second-year students: aOR: 1.18, 95% CI: 1.01–1.38). As for politics, only students that preferred to not disclose their orientation had higher odds of hesitancy compared to moderate respondents (aOR: 1.26, 95% CI: 1.08–1.49). On the other hand, while higher perceived COVID-19 severity and concern for the emergency were negatively associated with vaccine hesitancy (aOR: 0.89, 95% CI: 0.85–0.94, and aOR: 0.93, 95% CI: 0.89–0.98, respectively), higher susceptibility to COVID-19 did not show any relationship. The lowest odds of vaccine hesitancy were found for a 1-unit increase in confidence in vaccine safety (aOR: 0.56, 95% CI: 0.52–0.61) and effectiveness (aOR: 0.79, 95% CI: 0.73–0.86). In addition, wearing masks indoors or outdoors more frequently was negatively associated with vaccine hesitancy (aOR: 0.94, 95% CI: 0.89–0.99, and aOR: 0.92, 95% CI: 0.89–0.96, respectively). By contrast, age, nationality, finances, past COVID-19 infection, performing hand hygiene, or maintaining physical distancing more often did not seem to be predictors of the outcome.

The subgroup analysis performed on healthcare students yielded results similar to the main analysis, with the only exceptions of politics and mask wearing indoors that were no longer significant (Table S1). Specifically, like the main analysis, being surveyed during April–May, being male, and having a lower academic level were found to be positive associated with vaccine hesitancy, whereas higher levels of perceived COVID-19 severity, concern for the emergency, confidence in vaccine safety and effectiveness, and self-reported adherence to mask wearing outdoors were negatively associated with the outcome. Additionally, no association was found for gender, nationality, finances, past COVID-19 infection, performing hand hygiene, or maintaining physical distancing more often.

## 4. Discussion

Given that attitudes towards vaccinations have been shown to change over time [25], monitoring the vaccine hesitancy phenomenon and its determinants is universally recognized as a fundamental strategy for addressing any potential concern [26,27]. In this study, we found that, between March and June 2021, intention to accept vaccination against COVID-19 ranged from 71% to 78%, proportions that are lower than the 86% and 92% previously reported among Italian college students in April 2020 [20] and January 2021 [21], and midway between the 57% and 92% found in May 2021 [22] in a study that examined vaccination intention for viral vector and mRNA vaccines, respectively. Hence, these data may suggest that vaccination intention in this population subgroup has changed since the beginning of the vaccination campaign or, because we considered anyone who was not 100% willing to take the vaccine to be hesitant, the discrepancy with previous studies may also depend on how the outcome was measured, as already described [12]. However, despite these proportions being higher than those reported in other countries [14,15,19] or among the Italian adults aged 18–28 years (data not shown), our results likely confirm that an appreciable number of Italian college students have some hesitancy towards COVID-19 vaccination. For this reason, coordinated efforts are needed to address concerns and achieve optimal vaccine uptake in this subgroup [28]. Specifically, close attention should be paid to the development of effective and coherent communication strategies [29]. It may not be a coincidence that an intense media debate around adverse reactions to the Vaxzevira (AstraZeneca AB, Södertälje, Sweden) vaccine took place between March and April 2021 [30] and that we observed a higher likelihood of hesitancy during April–May 2021. Considering that confidence in vaccine safety was the strongest predictor of vaccination intention and that, as already indicated by other studies [15,31,32], safety and efficacy issues were among the most frequently reported reasons for hesitancy, the importance of limiting disinformation on COVID-19 vaccines to promote their public acceptance is further confirmed [29].

Among the factors explored, a few findings contrasted with the literature. Whereas it has been reported that either female respondents are less likely to intend to vaccinate [12,15] or there is no association with gender [21,33,34], in our study male participants were more likely to be hesitant. In addition, while in the general population different rates of hesitancy have been found according to the country studied [12], we did not detect any difference in terms of nationality, probably reflecting a mitigation of attitudes due to all our respondents being exposed to the same Italian environment. Conversely, our students seemed to be receptive to the scientific recommendation that natural immunity should not exempt an individual from vaccination [35]; higher levels of hesitancy were not observed in respondents reporting a past COVID-19 infection. As for finances and political ideology, two other well-documented determinants of vaccine-related attitudes and behaviors [12,25,36,37], in the whole sample we found an association for the latter only. It is difficult to hypothesize why those students who preferred not to disclose their political position were more likely to be hesitant; for example, these students may not have had a clear opinion, or they may not have wished to report it voluntarily, or a mixture of both. However, since the ongoing political debate around COVID-19 vaccinations is still intense [38] and the association was found in the main analysis only, a deeper understanding of the relationship between politics and vaccination intention in young adults is warranted.

With regard to education, the fact that a greater willingness to receive the vaccine was found among students attending healthcare curricula is comforting, given the clinical implications of a potential hesitancy among healthcare workers and students who are frequently in contact with high-risk patients, but it also suggests that strategies aimed at increasing health literacy among young people should be developed [39]. The importance of these interventions may be further confirmed by the fact that, while we do not detect any evidence relating to the age of respondents—probably because the age range investigated was narrow—students at the beginning of their academic career were more likely to be hesitant, as reported in France [15]. Therefore, implementing tailored approaches in the university setting during the early years of students’ education could improve vaccination compliance among young people, not only in the case of COVID-19, but also for other infectious diseases, such as measles or human papillomavirus, whose immunization rates are currently sub-optimal in this subgroup [40,41,42].

COVID-19 risk perception is another factor that has been shown to modulate attitudes and behaviors [43]. In our study, we found that the raw judgements of concern, likelihood of infection, and disease severity changed over time, in line with the dynamic nature of the process [44]. Additionally, our data showed that (i) participants did not feel they were at particularly high risk of infection and (ii) that this perception was not a strong predictor of vaccine hesitancy, suggesting that the vaccine decision-making process is affected by considerations of both personal and societal health benefits [4]. Within this context, implementing non-pharmaceutical interventions to limit the spread of the virus and protect others has been a key message of many communication campaigns across the world, including Italy [45]. Despite a few decreasing trends, we found that self-reported adherence to the guidelines was overall quite high, especially for mask wearing, both indoors and outdoors. Moreover, in the main analysis, compliance with these two recommendations seemed to be related to vaccine acceptance, probably because there are factors that are in common, such as having prosocial values and trust in science [46]. Therefore, communication strategies focusing on restoring public confidence in health authorities and helping people understand why recommended measures are useful for them and their community could simultaneously increase vaccine acceptance and adherence to recommended precautions [46].

Lastly, freedom to choose which vaccine to receive, and its association with hesitancy, has been hypothesized by a few authors [47], but the issue is still under-investigated. In our study, the main drivers of choice were comparable to those found among Japanese people in February 2021 [48], except for the country of vaccine production, which we found replaced by vaccine type. This difference may relate to when the various surveys were conducted; unlike the Japanese respondents, our students were exposed to media reports in March and April 2021, alleging a worse performance for Vaxzevira (AstraZeneca AB, Södertälje, Sweden) [29], the most well-known viral-vector vaccine. However, we did not investigate the effect of the freedom to choose the vaccine on vaccination intention. Further research on this is needed, as its impact may differ across countries and change over time, depending on pandemic conditions and local perceptions of vaccines [49]. Furthermore, if restricting freedom of choice is proven to increase vaccination hesitancy, effective communication strategies should be devised to change attitudes to particular vaccines [49]. Otherwise, or in addition, there should be a comprehensive evaluation of the ethical considerations that may arise as a consequence of allowing people to choose which vaccine they receive as a means of increasing vaccine uptake [49].

This study has some limitations. Firstly, the cross-sectional design hindered the opportunity to draw causal conclusions between vaccination intention and the associated factors. Secondly, since the participants of this study were recruited among those attending in-person activities at the main campus and participating in the SARS-CoV-2 screening campaign, it is possible that, despite the good response rate we achieved, our sample may be not entirely representative of Sapienza University students. Furthermore, since respondents were asked to fill out the questionnaire while waiting to be tested, the survey was constrained to a reduced number of questions, limiting the scale of the investigation. In this regard, we did not explore a few aspects that may be associated with vaccine hesitancy [4], such as knowledge, vaccine literacy, and social media influences. They are interconnected factors that make the individuals able to discern accurate information from misinformation [4]. Within this context, given their frequent engagement in social networks, students may be particularly exposed to false and misleading information regarding COVID-19 vaccinations and, since they may not be able to successfully manage the confusion coming from the infodemic, further analyses on the topic should be conducted. Nevertheless, to the best of our knowledge, this is the first study to track hesitancy towards SARS-CoV-2 vaccines during the first months of the vaccination campaign in a large sample of Italian university students. In addition, we were able to analyze student attitudes, risk perceptions, and adherence to recommended precautions in relation to vaccination intention, providing data that may support policymakers in developing effective communication strategies for the promotion of vaccine uptake.

## 5. Conclusions

During the first months of the vaccination campaign, we found that around one in four students had some degree of hesitancy towards COVID-19 vaccination. Additionally, vaccine hesitancy changed over time and in relation to several factors, including confidence in vaccine effectiveness and safety, risk perception of COVID-19, and education level. Since university students are a good target for intervention campaigns, as they are still undergoing education and may be open to a change in behavior, additional efforts to increase their awareness and engagement, restore confidence in health authorities, and limit disinformation regarding the vaccines should be made, especially in the university setting where students’ education is the main focus.

## Figures and Tables

**Table 1:** Students’ sociodemographic characteristics by survey period. Results are expressed as mean (standard deviation, SD), median (interquartile range, IQR), or frequency (percentage).

		Survey Period			*p*-Value *
		#1 1 March– 12 March 2021 N = 1910	#2 12 April– 9 May 2021 N = 1720	#3 10 May– 30 June 2021 N = 1739	
Age (years)					0.11
	Mean (SD)	23.5 (4.7)	23.4 (4.5)	23.5 (4.0)	
	Median (IQR)	22 (21–25)	22 (21–25)	23 (21–25)	
Gender					0.007
	Female	1130 (59.2)	1054 (61.3)	1117 (64.2)	
	Male	780 (40.8)	666 (38.7)	622 (35.8)	
Nationality					<0.001
	Italian	1744 (91.3)	1533 (89.1)	1490 (85.7)	
	Non-Italian	166 (8.7)	187 (10.9)	249 (14.3)	
Area of study					
	Healthcare	574 (30.1)	613 (35.6)	356 (20.5)	<0.001
	Science	544 (28.5)	421 (24.5)	582 (33.5)	
	Other	792 (41.5)	686 (39.9)	801 (46.1)	
Year of study					<0.001
	First or second	870 (45.6)	791 (46.0)	689 (39.6)	
	Third or above	1040 (54.4)	929 (54.0)	1050 (60.4)	
Finances					0.047
	I have many difficulties	101 (5.3)	91 (5.2)	107 (6.2)	
	I have some difficulties	453 (27.3)	473 (27.5)	434 (25.0)	
	Managing well enough	949 (49.7)	825 (48.0)	881 (50.7)	
	Managing very well	407 (21.3)	331 (19.2)	317 (18.2)	
Politics					0.009
	Moderate	1022 (53.5)	853 (49.6)	837 (48.1)	
	Strongly left-wing	237 (12.4)	200 (11.6)	214 (12.3)	
	Strongly right-wing	13 (0.7)	22 (1.3)	21 (1.2)	
	I prefer not to answer	638 (33.4)	645 (37.5)	667 (38.4)	

* Pearson’s chi-squared test for categorical variables and Kruskal–Wallis test for continuous variables.

**Table 2:** Students’ COVID-19 experience, risk perceptions, attitudes towards SARS-CoV-2 vaccination, and self-reported adherence to precautionary measures by survey period. Results are expressed as mean (standard deviation) or frequency (percentage).

		Survey Period			
		#1 1 March– 12 March 2021 N = 1910	#2 12 April– 9 May 2021 N = 1720	#3 10 May– 30 June 2021 N = 1739	*p*-Value *
Perceived susceptibility to COVID-19		4.86 (2.2)	4.50 (2.3)	4.00 (2.3)	<0.001
Perceived COVID-19 severity		7.76 (1.7)	7.53 (1.9)	7.34 (2.1)	<0.001
Concern about the COVID-19 emergency		7.95 (1.8)	7.58 (2.0)	7.21 (2.2)	<0.001
COVID-19 infection					<0.001
	No infection	1842 (96.4)	1614 (93.8)	1624 (93.4)	
	Asymptomatic	13 (0.7)	20 (1.2)	34 (2.0)	
	Mild symptoms	40 (2.1)	65 (3.8)	49 (2.8)	
	Moderate/severe symptoms	15 (0.8)	21 (1.2)	32 (1.8)	
Vaccine hesitancy					<0.001
	No hesitancy	1489 (78.0)	1246 (72.4)	1237 (71.1)	
	Some degree of hesitancy	421 (22.0)	474 (27.6)	502 (28.9)	
Reasons for hesitancy (N = 1397) ^a^					0.382
	I don’t believe in the safety of the vaccines available to me to date	96 (22.8)	93 (19.6)	89 (17.7)	
	I don’t consider myself at risk	76 (18.1)	97 (20.5)	87 (17.3)	
	I am aware of serious reactions that occurred to relatives/acquaintances after receiving the COVID-19 vaccination	55 (13.1)	114 (24.1)	131 (26.1)	
	I don’t believe in the effectiveness of the vaccines available to me to date	67 (15.9)	31 (6.5)	34 (6.8)	
	I don’t trust the authorities that encourage COVID-19 vaccination	26 (6.2)	20 (4.2)	25 (5.0)	
	I prefer getting natural immunity to COVID-19	13 (3.1)	26 (5.5)	37 (7.4)	
	I have already had COVID-19	8 (1.8)	21 (4.4)	14 (2.8)	
	I am suffering from a clinical condition with contraindication to COVID-19 vaccination	7 (1.7)	8 (1.7)	16 (3.1)	
	A person and/or an authority I trust encouraged me not to get vaccinated against COVID-19	5 (1.2)	3 (0.6)	6 (1.2)	
	I don’t believe in any vaccine including the COVID-19 vaccine	3 (0.7)	0 (0.0)	2 (0.4)	
	Other reasons	65 (15.4)	61 (12.9)	61 (12.2)	
Confidence in vaccine safety		8.05 (1.7)	7.82 (1.7)	7.54 (1.9)	<0.001
Confidence in vaccine effectiveness		7.85 (1.6)	7.9 (1.7)	7.69 (1.9)	0.054
Adherence to mask wearing indoors		9.19 (1.4)	9.13 (1.5)	8.90 (1.8)	<0.001
Adherence to mask wearing outdoors		8.67 (2.0)	8.57 (2.1)	8.19 (2.3)	<0.001
Performing hand hygiene		8.55 (1.7)	8.72 (1.8)	8.57 (2.0)	<0.001
Maintaining physical distancing		7.87 (1.8)	7.75 (2.1)	7.61 (2.1)	0.025

COVID-19: coronavirus disease 2019. ^a^ Total number of respondents. * Pearson’s chi-squared test for categorical variables and Kruskal–Wallis test for continuous variables.

**Table 3:** Students’ attitudes towards vaccination against SARS-CoV-2 by survey period (optional questions added to the questionnaire from 12 April 2021). Results are expressed as mean (standard deviation) or frequency (percentage).

		Survey Period		
		#2 12 April– 9 May 2021 N = 1720	#3 10 May– 30 June 2021 N = 1739	*p*-Value *
Prefer to choose which vaccine to take ^a^		6.76 (3.0)	6.99 (3.0)	0.005
Vaccine feature considered in the choice of vaccine ^a^				0.613
	Country of production of the vaccine or of the pharmaceutical company	60 (3.8)	77 (4.4)	
	Technology used or type of vaccine (mRNA, viral vector, etc.)	318 (20.4)	382 (22.0)	
	Higher efficacy in trials	391 (25.0)	401 (23.2)	
	Higher safety in trials	350 (22.4)	372 (21.4)	
	Fewer side effects reported as personal experiences	407 (26.0)	465 (26.8)	
	Other features	38 (2.4)	41 (2.4)	

^a^ Total number of respondents = 3302. * Pearson’s chi-squared test for categorical variables and Wilcoxon rank-sum test for continuous variables.

**Table 4:** Multivariable logistic regression model for COVID-19 vaccine hesitancy among the students surveyed between 1 March and 30 June 2021, Sapienza University of Rome.

		COVID-19 Vaccine Hesitancy	
		OR (95% CI)	*p*-Value
Survey period			
	#1 (1 March–12 March 2021)	Ref.	
	#2 (12 April–9 May 2021)	1.25 (1.04–1.50)	0.020
	#3 (10 May–30 June 2021)	0.99 (0.82–1.20)	0.933
Age (years)		1.01 (0.99–1.03)	0.164
Gender			
	Female	Ref.	
	Male	1.39 (1.18–1.64)	<0.001
Nationality			
	Italian	Ref.	
	Non-Italian	1.23 (0.97–1.57)	0.083
Area of study			
	Healthcare	Ref.	
	Science	1.33 (1.08–1.64)	0.007
	Other	1.40 (1.16–1.70)	<0.001
Year of study			
	Third or above	Ref.	
	First or second	1.18 (1.01–1.38)	0.049
Finances			
	Having some or many difficulties	Ref.	
	Managing well enough or very well	1.07 (0.91–1.27)	0.401
Politics			
	Moderate	Ref.	
	Strongly left-wing	1.00 (0.77–1.29)	0.999
	Strongly right-wing	1.10 (0.54–2.26)	0.788
	Prefer not to answer	1.26 (1.08–1.49)	0.004
Perceived susceptibility to COVID-19		1.03 (0.99–1.06)	0.152
Perceived COVID-19 severity		0.89 (0.85–0.94)	<0.001
Concern about the COVID-19 emergency		0.93 (0.89–0.98)	0.003
COVID-19 infection			
	No	Ref.	
	Yes	1.08 (0.78–1.49)	0.639
Confidence in vaccine safety		0.56 (0.52–0.61)	<0.001
Confidence in vaccine effectiveness		0.79 (0.73–0.86)	<0.001
Adherence to mask wearing indoors		0.94 (0.89–0.99)	0.013
Adherence to mask wearing outdoors		0.92 (0.89–0.96)	<0.001
Performing hand hygiene		0.98 (0.94–1.03)	0.425
Maintaining physical distancing		1.00 (0.96–1.05)	0.994

OR: Odds Ratio. CI: Confidence Interval. COVID-19: Coronavirus disease 2019.

## Data Availability

The data presented in this study are available on request from the corresponding author.

## Supplementary Materials

The following are available online at www.mdpi.com/article/10.3390/vaccines9111292/s1, Table S1: Multivariable logistic regression model for COVID-19 vaccine hesitancy among the healthcare students surveyed between 1 March and 30 June 2021, Sapienza University of Rome (N = 1543).
