# Supplementary material for: COVID-19 Vaccine Hesitancy among Italian University Students: A Cross-Sectional Survey during the First Months of the Vaccination Campaign
Source: Vaccines (Basel). 2021 Nov 7;9(11):1292. doi: 10.3390/vaccines9111292 (PMC8625887; doi:10.3390/vaccines9111292)
Supplement: Supplementary file 1 [file vaccines-09-01292-s001.zip › vaccines-1435170-supplementary.pdf]

Table S1. Multivariable logistic regression model for COVID-19 vaccine hesitancy among the healthcare students surveyed between March 1st and June 30th, 2021, Sapienza University of Rome (N=1543).

	COVID-19 Vaccine Hesitancy	
	OR (95% CI)	p-value
Survey period		
#1 (March 1st - March 12th, 2021)	Ref.	
#2 (April 12th - May 9th, 2021)	1.47 (1.02-2.13)	0.041
#3 (May 10th - June 30th, 2021)	0.24 (0.81-1.89)	0.326
Age (years)	1.04 (0.99-1.08)	0.050
Gender		
Female	Ref.	
Male	1.44 (1.01-2.05)	0.042
Nationality		
Italian	Ref.	
Non-Italian	1.05 (0.61-1.81)	0.856
Year of study		
Third or above	Ref.	
First or second	1.71 (1.23-2.37)	0.001
Finances		
Having some or many difficulties	Ref.	
Managing well enough or very well	0.88 (0.63-1.24)	0.461
Politics		
Moderate	Ref.	
Strongly left-wing	1.23 (0.71-2.11)	0.463
Strongly right-wing	1.40 (0.40-4.90)	0.595
Prefer not to answer	1.07 (0.76-1.50)	0.696
Perceived susceptibility to COVID-19	1.01 (0.94-1.08)	0.829
Perceived COVID-19 severity	0.87 (0.78-0.96)	0.008
Concern about the COVID-19 emergency	1.11 (1.01-1.23)	0.049
COVID-19 infection		
No	Ref.	
Yes	1.49 (0.81-2.72)	0.461
Confidence in vaccine safety	0.52 (0.44-0.61)	<0.001
Confidence in vaccine effectiveness	0.78 (0.67-0.92)	0.002
Adherence to mask wearing indoors	0.90 (0.81-1.01)	0.080
Adherence to mask wearing outdoors	0.90 (0.84-0.98)	0.013
Performing hand hygiene	0.92 (0.82-1.02)	0.132
Maintaining physical distancing	0.95 (0.87-1.05)	0.318

OR: Odds Ratio. CI: Confidence Interval. COVID-19: Coronavirus disease 2019.
